# Notes on the Scarlet Fever, as It Appeared in Green County, Ohio

**Published:** 1842-02

**Authors:** John Dawson


### Art. II.—*Notes on the Scarlet Fever, as it appeared in Green County, Ohio.* By John Dawson, M. D.

The season of '38 was characterized by a general drought, which in this region continued till the spring of '40. It was during this period the scarlet fever supervened. No case had been observed by the physicians for six or eight years previous.

The varieties of the disease, usually referred to by our standard authorities, could scarcely be recognized here. A much more correct division would have been into *simple* and *inflammatory*.

The simple variety was sometimes so slight in its invasion and progress, as scarcely to be noticed, either by the patient or his friends. In most cases no eruption made its appearance, and the fauces seldom required any other attention than a cleansing gargle. When, however, the action of the heart and arteries had become somewhat vehement, and the eruption was developed, when the tonsils and velum palati seemed to be suffering from inflammatory irritation, the disease was more serious, but always yielded readily to the proper treatment.

The *inflammatory* variety did not pass off in this way. Almost invariably it was ushered in with cerebral distress, such as violent pain in the head, or delirium, which, in several cases that come under my observation, produced convulsions that very speedily terminated in death. Its mode of attack differed widely from this in other cases. Vomiting and purging were occasionally the first symptoms of deranged health; sometimes purging alone, Irritation of the heart and arteries predominated in almost every case. Generally, the frequency of the pulse amounted to one hundred and twenty to the minute; and in some cases, it was so great from the commencement as to be indistinguishable. The fauces were the seat of an active inflammation, which involved the parotid glands, together with the muscles and lymphatics on the side of the neck. The tumefaction resulting from this produced in many cases death by strangulation. In other instances the inflammation extended to the brain or trachea, and hence some of the patients died with the symptoms of *Cephalitis* or *Laryngitis.*

*Prognosis.*—Generally speaking, the prognosis in the inflammatory variety was bad. Parents who had five or six children sometimes lost half, and others all, in as many days. In most instances where I observed recovery to take place, the individuals had been attacked with vomiting in the commencement. But if this was accompanied with purging, the patient invariably died. Purging by itself was little less fatal.

*Treatment.*—In the simple variety, this has already been anticipated. I believe such cases will get well under any plan of treatment. It is in the inflammatory form of the disease that we have the *opprobrium medicorum.* Here I had some cases in which I witnessed the inefficiency of every plan of treatment. I might relate a case in which the patient seemed literally inflamed from head to foot. The disease run its course, and terminated fatally, notwithstanding the employment of a bold and vigorous practice. Cases like this produced skepticism in the minds of some physicians relative to our power of controlling this disease in any form. But unwilling to subscribe to this sentiment, I will briefly relate the means which I believe saved some from an untimely grave. Of these, blood-letting was among the most efficient. This remedy was most valuable when the blood was suffered to flow in a full stream from a large orifice, until there was a decided impression made on the action of the heart and arteries. In some cases, this had to be repeated several times before the momentum of the circulation was completely controlled,

Next in importance to blood-letting, was the application of cold water to the skin. Nothing contributed more to lessen the exalted temperature, abate the febrile orgasm, and arrest the inflammatory diathesis of the whole system, than the application of cold water to the surface.

The water was most successfully applied by sponging and ablutions. We commenced with it at a temperature but little below that of the skin, and gradually diminished it until all the therapeutic effects of the remedy were obtained. *Purgatives* seemed to fulfil no other indication than just to keep open the first passages. Carried farther than this, they invariably did harm. But for accomplishing this object, calomel, from its soothing influence upon the irritated mucous surface of the bowels, was found to be the best. *Diaphoretics* did no good, except in the declension of the disease. *Capsicum*, so much lauded by some writers, was prejudicial, when administered internally—tending to increase the already over-excited condition of the system. Emollient cataplasms to the side of the neck, when the latter was much tumified, seemed to answer much better than blisters. They were always required, and seldom failed to mitigate the sufferings of the patient.

This is a condensed history of scarlatina, as it made its appearance with us. But I am informed by physicians from various parts of the state, that its violence and the mortality it occasioned in different regions were by no means uniform. In some places, it was similar to what it was here, whilst in others, it was altogether mild, requiring but little if any attention from the physician.

